# Optimisation of an *in vitro* human cardiovascular model on-a-chip for toxicological assessment of nicotine delivery products

**DOI:** 10.3389/ftox.2024.1395670

**Published:** 2024-06-13

**Authors:** Fiona Chapman, Luuk de Haan, Linda Gijzen, Wouter Strijker, Edgar Trelles Sticken, Sarah Jean Pour, Roman Wieczorek, Florian Haberstroh, Sandra Otte, Thomas Nahde, Liam Simms, Matthew Stevenson

**Affiliations:** ^1^ Imperial Brands PLC, Bristol, United Kingdom; ^2^ Mimetas BV, Oegstgeest, Netherlands; ^3^ Reemtsma Cigarettenfabriken GmbH, An Imperial Brands PLC Company, Hamburg, Germany

**Keywords:** cardiovascular disease, cigarette, co-culture, heated tobacco, electronic nicotine delivery system

## Abstract

**Background:**

Smoking cigarettes is a cause of serious diseases in smokers, including cardiovascular disease. Through a pathway of endothelial dysfunction, lipid infiltration, macrophage recruitment and vascular remodeling, atherosclerosis is fundamental in the development of most cardiovascular diseases. There is an increasing number of next-generation products (NGP) which provide potentially reduced harm forms of nicotine delivery to adult smokers. This study aimed to optimise an *in vitro* cardiovascular model to assess such products. Human Coronary Artery Endothelial Cells (HCAECs) were cultured on an OrganoPlate^®^2-lane chip (Mimetas BV) combined with THP-1 monocytes under flow conditions.

**Methods:**

An aqueous aerosol extract from the 1R6F reference cigarette was compared with two categories of NGP, (a heated tobacco product (HTP) and an electronic nicotine delivery system (ENDS)), to assess relative effects on select atherogenic endpoints (oxidative stress, monocyte adhesion, ICAM-1 expression, and inflammatory markers). Following exposure of THP-1 monocytes with the aqueous extracts, the resulting conditioned medium was then added to the HCAEC vessels.

**Results:**

1R6F was consistently the most potent test article, eliciting observed responses at 4x lower concentrations than applied for both the HTP and ENDS. The HTP was more potent than the ENDS product across all endpoints, however, all test articles increased monocyte adhesion. ICAM-1 did not appear to be a main driver for monocyte adhesion, however, this could be due to replicate variability. Upon comparison to an extract-only control exposure, THP-1-medium pre-conditioning was an important mediator of the responses observed.

**Conclusion:**

In conclusion, the data suggests that the NGP extracts, containing primary aerosol chemical constituents exhibit a marked reduction in biological activity in the early key events associated with atherogenesis when compared to a cigarette, adding to the weight of evidence for the tobacco harm reduction potential of such products.

## Introduction

Smoking is a cause of serious diseases, including lung cancer, emphysaema and cardiovascular disease (United States Surgeon General, 2010; International Agency for Research on Cancer, 2012; US Department of Health and Human Services, 2014; Ding et al., 2019). Atherosclerosis is reportedly responsible for the development of most cardiovascular diseases and develops through a pathway of endothelial dysfunction, lipid infiltration, macrophage recruitment and vascular remodelling (Björkegren and Lusis, 2022; Fearon et al., 2013; Kaplan et al., 2017). Endothelial dysfunction can be caused by a number of factors, including exposure to toxins such as those in cigarette smoke, high blood pressure, diabetes, hyperlipidemia and regional mechanical factors (Insull, 2009; Fearon et al., 2013; Rafieian-Kopaei et al., 2014; Libby, 2017; Wang et al., 2022); this leads to the subsequent infiltration of lipids into the vascular intima (Björkegren and Lusis, 2022). Coupled with this, oxidative stress drives the expression of adhesion molecules such as immunoglobulin cell adhesion molecule-1 (ICAM-1) and vascular cell adhesion molecule-1 (VCAM–1), and the subsequent recruitment and migration of monocytes to the site (Habas and Shang, 2018; Björkegren & Lusis, 2022). These circulating monocytes are critical players within innate immunity, having a crucial role in the initiation of inflammatory responses (Chen et al., 2022). These monocytes subsequently differentiate to macrophages, which take up oxidised low-density lipoprotein (LDL) in the vessel intima; these cells undergo necrotic processes, which among other events, leads to plaque formation and eventually rupture, at which point thrombosis occurs (Björkegren and Lusis, 2022).

Combusting tobacco produces smoke containing approximately 7,000 chemicals, around 100 of which are classified by public health experts as harmful and potentially harmful constituents (HPHCs) (USDHHS, 2010). Tobacco harm reduction (THR) is the concept of providing adult smokers, who do not wish to quit nicotine and would otherwise continue to smoke, with potentially reduced harm forms of nicotine delivery (O’Leary and Polosa, 2020). There are a number of options available with THR potential for adult smokers, including next-generation product (NGP) innovations such as electronic nicotine delivery systems (ENDS) and heated tobacco products (HTP). Both of these product categories involve generation and inhalation of nicotine-containing aerosols, which have been demonstrated to contain fewer and lower levels of toxicants compared to cigarette smoke (Forster et al., 2018; Bentley et al., 2020; Rudd et al., 2020; Chapman et al., 2023). Furthermore, nicotine delivery products are proposed to sit on a relative risk (of exposure to toxicants) scale, where cigarettes are associated with substantially higher risk than NGP, and nicotine replacement therapies pose the lowest risk (McNeill and Munafò, 2013; Abrams et al., 2018; Zeller, 2019; Murkett et al., 2020). Within the relative risk scale, ENDS are generally associated with lower numbers and levels of toxicants within their aerosols compared to HTP, and this is reflected in their relative *in vitro* toxicity profiles; further to this, NGP samples have been associated with substantially reduced toxicological outcomes compared to cigarettes (Jaunky et al., 2018; Hattori et al., 2020; Giebe et al., 2021; Simms et al., 2022). Due to the relative nascency of NGP such as ENDS and HTP compared to traditional tobacco products such as cigarettes, the potential long-term effects on cardiovascular health with such products is yet to be fully defined (Tsai et al., 2020).

With the increasing availability of such novel nicotine delivery products, it is important to gain an understanding of their potential to induce the processes involved in smoking-related diseases, such as cardiovascular disease, and also to further characterise their THR potential compared to cigarettes. Several *in vitro* models have been developed to model cardiovascular endpoints, including measures of cardiac cell functionality, cardiovascular wound healing and also models under different flow conditions to more closely mimic *in vivo* scenarios (Poussin et al., 2014; Poussin et al., 2018; Giebe et al., 2021; Simms et al., 2022). The availability of human cell-derived 3D vasculature models-on-chips further increases the *in vivo* relevance of the outcomes, which can also be coupled with immune components, for example, monocytes, to model the inflammatory processes involved in atherosclerotic development (Poussin et al., 2014; Poussin et al., 2020; de Haan et al., 2021; Ohashi et al., 2023). One such model is the OrganoPlate^®^ chip (Mimetas BV), which has previously been utilised for *in vitro* assessment of extracts derived from cigarette smoke and NGP aerosols (Poussin et al., 2018; Poussin et al., 2020; Ohashi et al., 2023). The system utilises a collagen matrix to guide the formation of vessels within the chip, which is available within 2-lane or 3-lane formats dependent on model configuration and endpoints being assessed, and the chip is cultured and maintained under flow conditions to more closely mimic an *in vivo* scenario. Particularly, the bidirectional flow achieved using the model, at around a shear stress of 1.6 dyn/cm^2^, mimics the turbulence and lower flow rate (<5 dyn/cm^2^) observed in regions prone to atherosclerotic lesions, i.e., at vessel bends and bifurcations (Vormann et al., 2018; Warboys et al., 2011; Poussin et al., 2018). To date, this model has only been used to assess a limited number of nicotine delivery product variants and has not been used to assess the *my*blu ENDS and Pulze and iD HTP specifically. Furthermore, this is the first study, to our knowledge, to compare the three product categories directly with this model.

A recently published adverse outcome pathway outlined a number of key events involved in atherosclerosis progression (Ohara and Ito, 2023), therefore associated endpoints were assessed within this study (1–4). Oxidative stress (1) has a known role in endothelial dysfunction; oxidised LDL invades the vessel intima, but oxidative species also play a role in cellular regulation and expression of immune cell recruitment molecules (Ohara and Ito, 2023). This was assessed using a glutathione depletion assay where levels will decrease with an increase in the presence of reactive oxidative species. Monocyte adhesion (2) is the result of expression of cell surface markers such as ICAM-1 (3) during endothelial dysfunction (Edwards and Thomas, 2017; Makwana et al., 2021; Ohashi et al., 2023), therefore these two endpoints were also assessed. Finally, to further characterise drivers of any contributing inflammatory responses to the respective test articles, a panel of inflammatory markers (4) released into the vessel medium during the 24 h exposure and present in the pre-conditioned exposure medium applied, was explored. To achieve this, Human Coronary Artery Endothelial Cells (HCAECs) were cultured on an OrganoPlate 2-lane chip (Mimetas BV) and exposed to medium pre-conditioned by exposing THP-1 monocytes to aqueous extracts from the smoke/aerosol of three nicotine delivery products (the 1R6F reference cigarette smoke, *my*blu™ ENDS aerosol and Pulze™ and iD™ HTP aerosol bubbled phosphate buffered saline (bPBS) solution). 1R6F and a range of PBS concentrations were initially applied to the system to elucidate appropriate doses for application in the main experiment and, along with the other endpoints, cell viability and barrier integrity were assessed. Following this, all test articles were tested with the model, and the responses to pre-conditioned medium were additionally compared to responses to the test articles applied directly to the vessel without medium pre-conditioning. The study also aimed to elucidate the relative effects of the three test articles with reference to their relative placements on the relative risk (of exposure to toxicants) scale for nicotine delivery products, as these particular test articles have not previously been applied to/compared in such a model.

## Methods

### Test articles, smoke/aerosol extract generation and dosimetry

Three nicotine delivery products were used in this study: 1) the 1R6F Kentucky reference cigarette (1R6F) (University of Kentucky, Kentucky, USA); 2) an ENDS product, the *my*blu pod-based system (Imperial Brands PLC); and 3) a HTP, the Pulze and iD stick heated tobacco system (Imperial Brands PLC). The *my*blu ENDS was of a tobacco flavour e-liquid variant, with a 1.6% nicotine concentration, and the system was as described by Rudd et al. (2020); the iD stick contained a reconstituted tobacco portion similar to the ‘Regular’ product as previously described and used in Chapman et al. (2022). The iD stick had a target specification of 4.6 mg nicotine/stick.

Aqueous phosphate buffered saline (PBS) (Sigma Aldrich, Germany) extracts of the aerosols generated from the respective products were created for application to the *in vitro* experimental system using the Vitrocell VC 10S-Type smoking robot. These extracts (termed bubbled PBS (bPBS)) were generated by bubbling the respective experimental products’ aerosols through three in-line impingers, each containing 10 mL PBS, which was then combined into 30 mL stock solutions. 1R6F smoke was generated according to the International Organization for Standardization (ISO, 20778:, 2018) formerly known as Health Canada Intense smoking regime 55 mL puff volume, 2 s puff duration, 30 s puff interval, bell-shaped puff profile, with ventilation blocking; the HTP bPBS was generated using a modified version of this regime, i.e., no ventilation blocking was applied. The ENDS aerosol was generated according to ISO 20768: 2018 (formerly known as the Cooperation Centre for Scientific Research Relative to Tobacco (CORESTA) Recommended Method N°81) (ISO, 20778:, 2018): 55 mL puff volume, 3 s puff duration, 30 s puff interval, square-wave puff profile. The pooled solution concentration for 1R6F = 1.8puffs/mL (54 puffs/30 mL) and the ENDS and HTP products 4.8puffs/mL (144 puffs/30 mL).

This extract was analysed to confirm smoke/aerosol trapping, using nicotine and eight carbonyls (formaldehyde, acetaldehyde, acetone, acrolein, propionaldehyde, crotonaldehyde, 2-butanone, n-butyraldehyde) as markers. These markers were selected based on those detailed by Buratto et al. (2018), and additionally, are present on regulatory HPHC lists (FDA, 2012); detailed methodology of their quantification in the PBS is described by Chapman et al. (2023).

### Cell culture

Two cell lines were used for the co-culture model, Human Coronary Artery Endothelial Cells (HCAECs) (PromoCell, C12221) and the human leukaemia monocytic cell line, THP-1, (ATCC, Tib-202). Prior to seeding, HCAECs were thawed and cultured in T-75 flasks (Corning, 431464U) using MV2 cell culture medium (Promocell, C22022) containing supplement mix (Promocell, C39226) and 1% Penicillin/Streptomycin (Sigma Aldrich, P4333), further referred to as MV2 medium. HCAECs were harvested at passage 5 by washing three times with PBS (Thermo Fisher Scientific, 70,013,016) and addition of 0.025% trypsin/ethylenediaminetetraacetic acid (EDTA, Lonza, CC-5012).

THP-1 cells were thawed and cultured upright in T-25 flasks (Corning, 353,009) using Roswell Park Memorial Institute (RPMI) 1,640 medium (Sigma Aldrich, R0883) supplemented with 10% fetal bovine serum (FBS), 1% Penicillin/Streptomycin and 1% GlutaMAX (Gibco, 35,050). Cells were maintained around 2 × 10^5^ - 5 × 10^5^cells/mL during culture.

### OrganoPlate 2-lane 96

The OrganoPlate 2-lane 96 (MIMETAS, 9605400B, Oegstgeest, Netherlands), consisting of 96 microfluidic chips with, respectively, 9 mm and 12.2 mm long gel and perfusion channels (400 µm × 220 µm (w x h)), was used in this study. A 4 mg/mL extracellular matrix (ECM) gel, consisting of 100 mM N-(2-Hydroxyethyl)piperazine-N′-(2-ethanesulfonic acid) (HEPES) buffer, (Gibco, 15,630-056), 3.7 mg/mL NaHCO_3_ (Sigma Aldrich, S5761) and 5 mg/mL collagen I (Cultrex, 3447-020-01), was prepared and 2 µL was dispensed in the gel inlets of the microfluidic chips. Plates were incubated at 37°C to allow polymerization of the ECM gel after which 50 µL of Hank’s Balanced Salt Solution (HBSS) (Thermo Fisher Scientific, 14175095) was added to the gel inlets to prevent dehydration. In addition, 50 µL of HBSS was added to the observation windows to prevent evaporation and increase optical clarity. Plates were placed in the incubator (37°C, 5% CO_2_) overnight until cell seeding.

**FIGURE 1:**
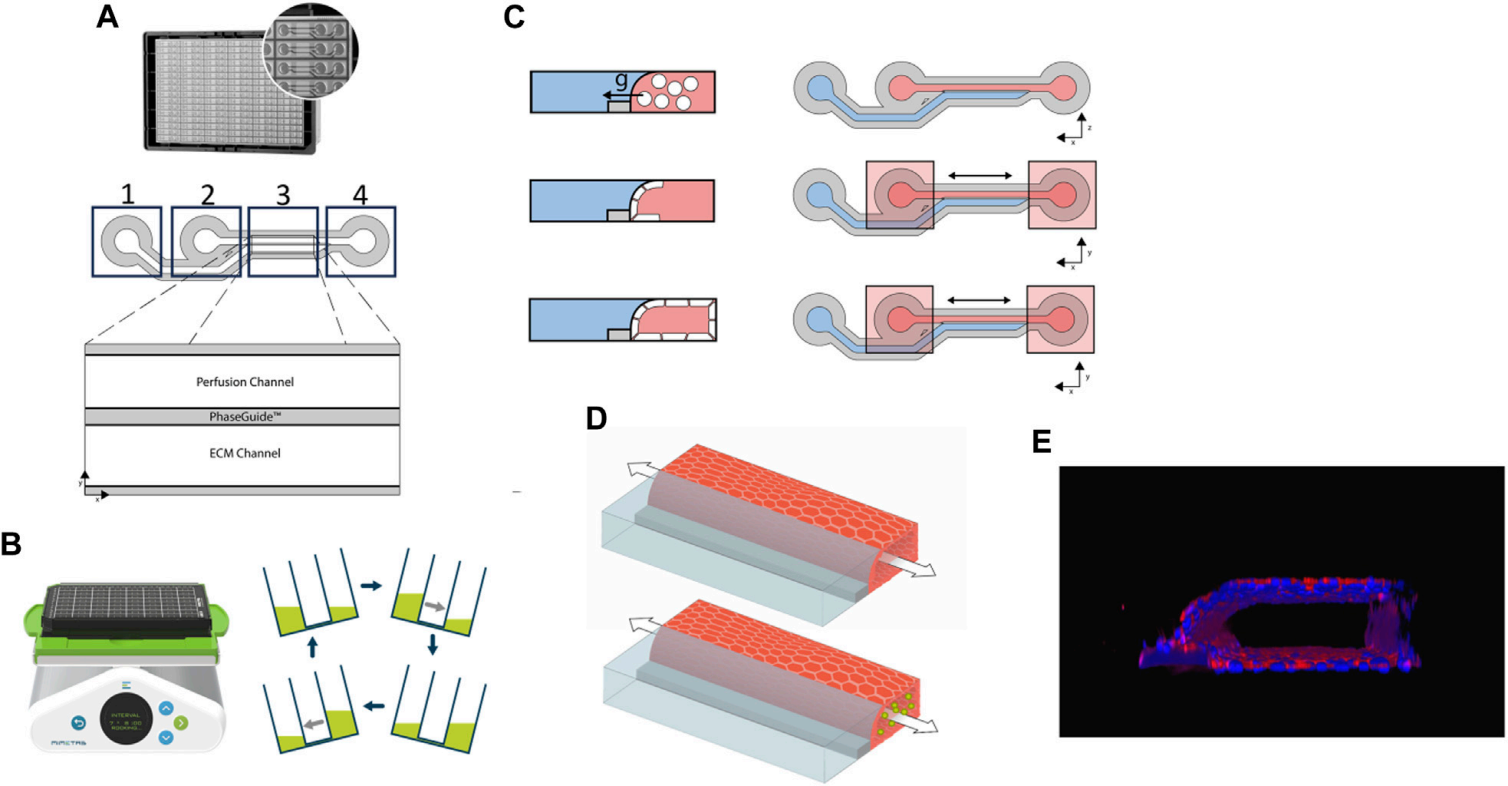
**(A)**. Image and representative diagram of the 2-lane OrganoPlate. Boxes 1, 2 and 4 mark where reagents/cells can be added to the respective parts of the chip. *x* and *y*-axes show the chip orientation on the plate; **(B)**. Rocker with plate and diagrammatic representation of the direction and gradient of media flow; **(C)**. Diagrammatic representation of the formation of the cell layer following seeding of the human coronary artery endothelial cells (HCAECs) (cells shown in white) within the 2-lane OrganoPlate. Red areas represent the perfusion channel, blue areas the extracellular matrix (ECM) and grey the plate PhaseGuide. Bidirectional arrows represent the direction of flow and *x* and *y*-axes the orientation; g = gravity. **(D)**. Diagrammatic representation of the cell culture within the OrganoPlate 2-lane. The arrow represents the bidirectional flow of medium/suspension cells; green circles represent THP-1 cells; the endothelial cell vessel is represented in red and the extracellular collagen matrix in blue; **(E)**. Image of the 3D HCAEC vessel stained with CD31.

Harvested HCAEC cells were counted using Trypan Blue (1:1) and the LUNA-II automated cell counter (Logos Biosciences, L40002), spun down (200 x g, 5 min) and resuspended at a density of 1 × 10^6^ cells/mL using MV2 medium. 2µL of cell suspension was dispensed in perfusion inlets of microfluidic chips, followed by 50 µL of MV2 medium. Plates were incubated at 37°C, 5% CO_2_ on their side for 2 h to allow cell adherence to the ECM gel. Next, 50 µL MV2 medium was added to perfusion outlets and plates were placed on a rocking interval platform (MIMETAS, MI-OFPR-L) set at 7° with 8-min intervals to induce gravity-driven flow. Figure 1 describes the plate set-up.

### Medium pre-conditioning using THP-1 monocytes for HCAEC exposure

Prior to exposure within the OrganoPlate system, cell culture medium was pre-conditioned with the respective test articles at the concentrations detailed in Table 1 using THP-1 cells to model the presence of systemic cellular responses, for example, the generation of cytokines, within the co-culture. The THP-1 cells were thawed and cultured for 48 h prior to being spun down and resuspended at a concentration of 2 × 10^6^ cells/mL for a 2 h exposure. Harvested conditioned medium was then frozen prior to use in the main study.

**TABLE 1:** Concentrations of bubbled phosphate buffered saline (bPBS) solution (%) applied to the cell culture medium for respective treatments, along with the equivalent nicotine concentration present (based of quantification within the bPBS stock solution). ENDS: Electronic nicotine delivery system; HTP: Heated tobacco product.

Dose level	1R6F		HTP		ENDS	
	% bPBS	μg/mL nicotine	% bPBS	μg/mL nicotine	% bPBS	μg/mL nicotine
1	1.25	2.30	5	10.11	5	13.41
2	2.5	4.60	10	20.22	10	26.82
3	5.0	9.19	20	40.44	20	53.64

To understand the effect of pre-conditioning on the outcomes measured, a comparison was also made to the responses in the OrganoPlate where the top concentration of the respective test articles was added directly with no pre-conditioning step.

To initially assess maximal bPBS concentrations that could be applied to the cell cultures (to match pre-conditioning and co-culture exposure concentrations), a range of levels of PBS, including standard concentrations (i.e., 10% or below) and higher concentrations were added to 250 µL of 2 × 10^6^ cells/mL suspension in 48-well plates for 2 h (0, 2.5, 5, 10, 20, 40% PBS). Cell viability was determined using Trypan Blue staining (1:1 with cell suspension) and the LUNA automated cell counter.

Based on this assessment, it was decided that 20% would be the maximal applied concentration for the pre-conditioning step due to greater variability in outcomes following the 40% exposure (Supplementary Figure S1).

### Exposures

An initial dose range finding experiment was carried out to select appropriate, non-cytotoxic concentrations for the study presented here. Based on historical data, 1R6F was expected to be the most potent test article, and this was applied to the model in the first instance to assess cell viability, barrier integrity, oxidative stress, monocyte adhesion and ICAM-1 expression.

### Phase contrast imaging

Automated phase contrast imaging was performed on regular basis using the ImageXpress XLS Micro HCI system (Molecular Devices, San Jose, CA, USA).

### Barrier function measurement

Cell culture medium was replaced by fresh MV2 medium containing 0.5 mg/mL tetramethylrhodamine isothiocyanate (TRITC)-dextran (155kDa, Sigma Aldrich, T1287). First, 10 µL fresh MV2 medium was added to the gel inlets of the chips. Next, MV2 medium containing TRITC-dextran was perfused through the lumen of the endothelial vessel in the top channel (40 µL in the inlet and 30 µL in the outlet). Leakage of the dye from the lumen of the endothelial vessel into the adjacent extracellular matrix compartment was recorded using time-lapse imaging on an ImageXpress XLS Micro high content imaging (HCI) system (7 timepoints, 2 min intervals). Ratio between fluorescent signal in the endothelial vessel compartment (donor) and the ECM compartment (receiver) was calculated using FIJI (version 2 build 1.52d) and used to calculate apparent permeability (P_app_) values.

### Cell viability measurement using water-soluble tetrazolium salt

Cell viability was measured using the Cell Counting Kit-8 (Sigma Aldrich, 96992). The water-soluble tetrazolium salt (WST-8) reagent was diluted 1:11 in MV2 medium as recommended by the supplier. Cell culture medium was replaced with diluted WST-8 reagent. Cultures were incubated at 37°C, 5% CO_2_ on a rocking interval platform (8 min, 7° angle) for 30 min. After incubation, the plate was placed static for 2 min before absorbance was measured at 450 nm using a multi-well plate reader (TECAN Spark^®^, TECAN Life Sciences). Acquired absorbance values were corrected for chip dimensions and corresponding volumes.

### Glutathione measurement

Assessment of glutathione (GSH) levels within the cell system was carried out using monochlorobimane (mBCI, Sigma Aldrich, 69899-5 MG), which binds to GSH to produce a fluorescent conjugate with an absorption/emission of 394/490 nm. Endothelial vessels were incubated with fresh MV2 medium containing 125 μg/mL mBCl +1:1000 DRAQ5 (Abcam, ab108410) at 37°C, 5% CO_2_ on a rocking interval platform (8 min, 7° angle) for 30 min after which the chips were washed with HBSS. Fresh HBSS was added to the chips and cultures were imaged using an ImageXpress Micro Confocal HCI System (Molecular Devices). Z-series were captured to generate sum projections which were used to quantify the cumulative intensity of the fluorescent signal after background subtraction using FIJI. Number of endothelial nuclei was quantified using built-in features of FIJI; first, a rolling ball background correction was applied to improve the signal-to-noise ratio (Sternberg, 1983) after which an automated thresholding routine was performed followed by particle detection to quantify individual nuclei. Subsequently, the mean fluorescent intensity per nucleus was calculated.

Based on the expected window to observe effects on GSH depletion (i.e., presence of oxidative stress), this analysis was carried out 4 h following first exposure of the OrganoPlate model to the test articles. Sigma Aldrich, SML1083 was applied as a positive control at a concentration of 25 μM for the GSH assay.

### Monocyte adhesion assay

To assess monocyte adhesion, fresh monocytes (i.e., not those which had been previously exposed in the generation of the pre-conditioned media) were applied to the OrganoPlate. THP-1 monocytes were suspended at a density of 1 × 10^5^cells/mL in complete RPMI medium containing 0.5 μg/mL Calcein-AM (Life Technologies C3099, Carlsbad, CA, USA). After labelling for 15 min at 37°C, the monocytes were spun down (200 x g, 5 min) and resuspended in fresh MV2 medium at a density of 1 × 10^5^ cells/mL. Meanwhile, nuclei of the endothelial vessels were stained by incubating with 1:2000 Hoechst 33342 (Thermo Fischer Scientific, H3570) for 20 min at 37°C. The endothelial vessels were washed with fresh MV2 medium after which labelled THP-1 monocytes were added apically to the endothelial vessels. Cultures were incubated at 37°C and 5% CO_2_ for 15 min on a rocking interval platform (8 min, 7° angle) to allow for monocyte adhesion and cultures were washed twice with HBSS. Cultures were imaged using an ImageXpress Micro Confocal High-Content Imaging System. The number of endothelial nuclei were quantified in FIJI as described before. The number of adhering monocytes was quantified using a nearly identical method as described before, except a minimum size of 10 µm was selected.

### ICAM-1 expression

Cultures were fixed after the experiment using 3.7% paraformaldehyde (Sigma Aldrich, P6148) in HBSS (Sigma Aldrich, 55037C). After washing twice with HBSS, cultures were permeabilized for 10 min using 0.3% Triton X-100 (Sigma Aldrich, T8787) and incubated with blocking solution containing 2% FBS, 2% bovine serum albumin (BSA; Sigma Aldrich, A2153) and 0.1% Tween-20 (Sigma Aldrich, P9416) for 45 min. Next, cultures were washed with PBS containing 4% FBS and incubated overnight with anti-ICAM-1 primary antibody (1:100; R&D systems, BBA3). Cultures were then washed and incubated with donkey anti-mouse 647 secondary antibody (1:250; Life Technologies, A31571) for 30 min. Hoechst 33342 (1:2000) was used as a nuclear counterstain. All steps were performed at room temperature. For the representative image in Figure 1E, CD31 staining was carried out as above, however, the primary antibody, CD31 was added (1:20, Dako, M0823) followed by secondary antibody, goat anti-mouse 488 (1:250, Life Technologies, AF32723).

Images were acquired with an ImageXpress Micro Confocal High-Content Imaging System. Z-series were acquired, and sum projections were generated for quantification of the fluorescent signal. The mean fluorescent intensity was calculated in FIJI and corrected for the number of endothelial nuclei.

### Cytokine analysis

Culture medium was sampled after exposure and frozen at −80°C. Cytokine contents of sampled medium was assessed using a ProCartaPlex Inflammation 20-plex (Thermo Fisher Scientific, EPX200-12185-901). The following analytes were measured: GM-CSF, IFN-α, IFN-ɣ, IL-1α, IL-1β, IL-4, IL-6, IL-8 (CXCL8), IL-10, IL-12p70, IL-13, IL-17A, TNF-α, IP-10 (CXCL10), MCP-1 (CCL2), MIP-1α (CCL3), MIP-1β (CCL4), ICAM-1, CD62E (E-selectin) and CD62P (P-selectin). Medium samples were processed according to supplier’s protocol and cytokine contents were measured using MAGPIX^®^ (Luminex Corporation, Austin, TX).

### Statistical analyses

Nine chips/test conditions were used across 2 biological replicates (n = 9, N = 2). Data was normalised to respective controls for visualisation, and error bars plotted were standard deviation (SD) about the mean. Statistical significance was determined using a one-way analysis of variance (ANOVA) using GraphPad Prism version 9. Statistical significance is marked as follows: *p < 0.05, **p < 0.01, ***p < 0.001 and ****p < 0.0001.

A summary of the experimental timeline can be found in Table 2.

**TABLE 2:** Overview of study timeline and key experimental steps. ECM: Extracellular matrix; GSH: Glutathione; HCAECs: Human coronary artery endothelial cells; ICAM-1: Immunoglobulin cell adhesion molecule-1; mBCl: Monochlorobimane; PBS: Phosphate buffered saline solution.

Day −1	Collagen I ECM gel seeded into OrganoPlate
Day 0	HCAECs seeded against ECM gel
Day 3	Test article extracts added to cell culture medium (containing THP-1 cells) (pre-conditioning) (negative control = PBS)
Day 4	OrganoPlate culture exposed to - Preconditioned medium - Test article extracts (compound only controls) - Positive control, TNF-α - Positive control (GSH assay), ethacrynic acid
	4 h analysis GSH assessment (mBCl added to cultures)
Day 5	Fresh THP-1 monocytes added for adhesion assay
	24 h analyses - Inflammatory mediators (in medium samples) - Monocyte adhesion (immunofluorescent readout) - ICAM-1 expression (immunofluorescent readout)

## Results

### Trapped aerosol dosimetry

To confirm aerosol trapping, nicotine and eight select carbonyls (selected based on a list outlined by Buratto et al., 2018) were quantified within the respective bPBS stock solutions generated for the test articles. Nicotine was also used as a dosimetry marker. Out of the eight carbonyls, only formaldehyde was quantifiable within the ENDS bPBS (at a substantially lower level than measured for 1R6F), with the other carbonyls reported as below the limit of quantification (LOQ). For the HTP, all eight carbonyls were present in the bPBS, however levels were below/substantially below those seen for the 1R6F reference cigarette. Trapped nicotine was highest in the ENDS bPBS, followed by the HTP bPBS, then 1R6F bPBS. Based on the respective test article concentration ranges applied in the subsequent cell culture analyses, the ENDS and HTP bPBS therefore delivered greater nicotine levels than those applied for the 1R6F bPBS (Table 3). When the top concentrations added to the cell cultures were compared, levels of carbonyls were approximately equivalent between 5% 1R6F bPBS and 20% HTP bPBS. However, the 1R6F stock required fewer puffs per mL PBS than the NGP to achieve these final concentrations and upon comparison on a per puff basis, levels of carbonyls in the 1R6F smoke were 5–19 and 70 times higher than for the HTP and the formaldehyde in the ENDS sample respectively.

**TABLE 3:** Nicotine and carbonyl quantification in bubbled phosphate buffered saline (bPBS) solution extracts. ENDS: Electronic nicotine delivery system; HTP: Heated tobacco product.

Analyte (μg/mL)	1R6F	HTP	ENDS
Nicotine	183.7	202.2	268.2
Formaldehyde	7.57	1.09	0.29
Acetaldehyde	152.76	42.48	<LOQ
Acetone	20.38	4.02	<LOQ
Acrolein	1.12	0.56	<LOQ
Propionaldehyde	8.02	2.12	<LOQ
Crotonaldehyde	3.43	0.70	<LOQ
2-Butanone (MEK)	3.99	0.71	<LOQ
n-Butyraldehyde	2.55	1.47	<LOQ

### Dose range finding and initial model validation with 1R6F bPBS

**FIGURE 2:**
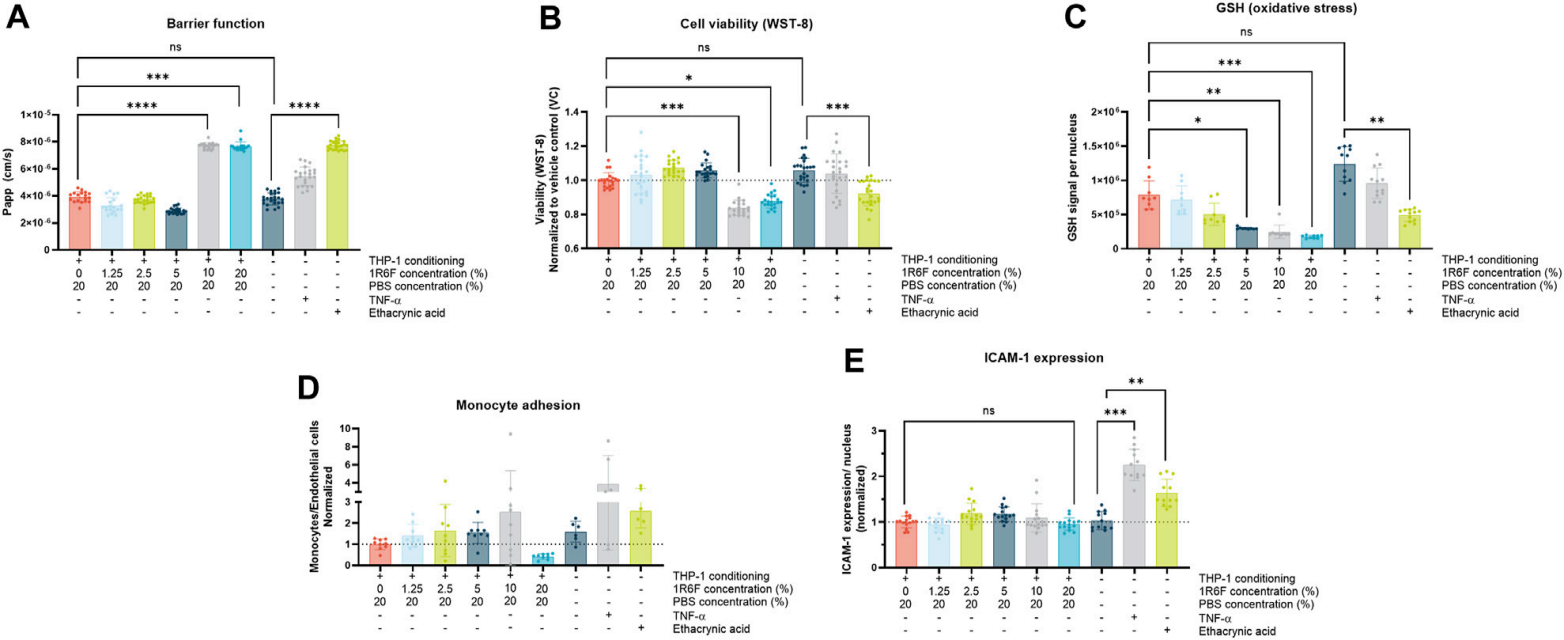
**(A)**. Barrier function; **(B)**. Endothelial cell viability; **(C)**. Glutathione (GSH) signal; **(D)**. Monocyte adhesion; and **(E)**. Endothelial ICAM-1 expression following exposure of the model to 1R6F-bPBS pre-conditioned medium and treated at the concentrations shown for 24 h (GSH measured at 4 h). Corresponding total PBS vehicle (20%), along with positive controls, ethacrynic acid and TNF-α are shown. Statistical significance was determined using a one-way ANOVA. Statistical significance is marked as follows: ns = not significant, *p < 0.05, **p < 0.01, ***p < 0.001 and ****p < 0.0001.

To determine appropriate concentrations to use in the main *in vitro* study, 1R6F was assessed as, based on previous studies, it was the most potent test article (Simms et al., 2022) (Figure 2). Additionally, two PBS control conditions were applied (20% and 40%) to elucidate the maximum bPBS stock concentration that could be applied with regards to the two less potent test articles (HTP and ENDS). At the 1R6F concentrations tested, barrier integrity (indicated by apparent permeability) declined significantly compared to PBS control at 1R6F concentrations of 10% and 20%; this effect was also observed at these concentrations upon assessment of cell viability. Dose-dependent increases in oxidative stress (indicated by depletions in GSH levels) were also observed for 1R6F. Up to 10% 1R6F bPBS, there appeared to be a dose-dependent increase in monocyte adhesion, however, due to high variability, this result was not statistically significant; the 20% 1R6F bPBS concentration induced a decrease, however, which may have been linked to reductions in cell viability and barrier integrity. Upon assessment of ICAM-1 expression, there were no significant increases upon application of the test articles, however positive controls TNF-α and ethacrynic acid induced significant increases, confirming the validity of the assay.

### Assessment of cigarette, ENDS and HTP aerosol bPBS

#### Oxidative stress

**FIGURE 3:**
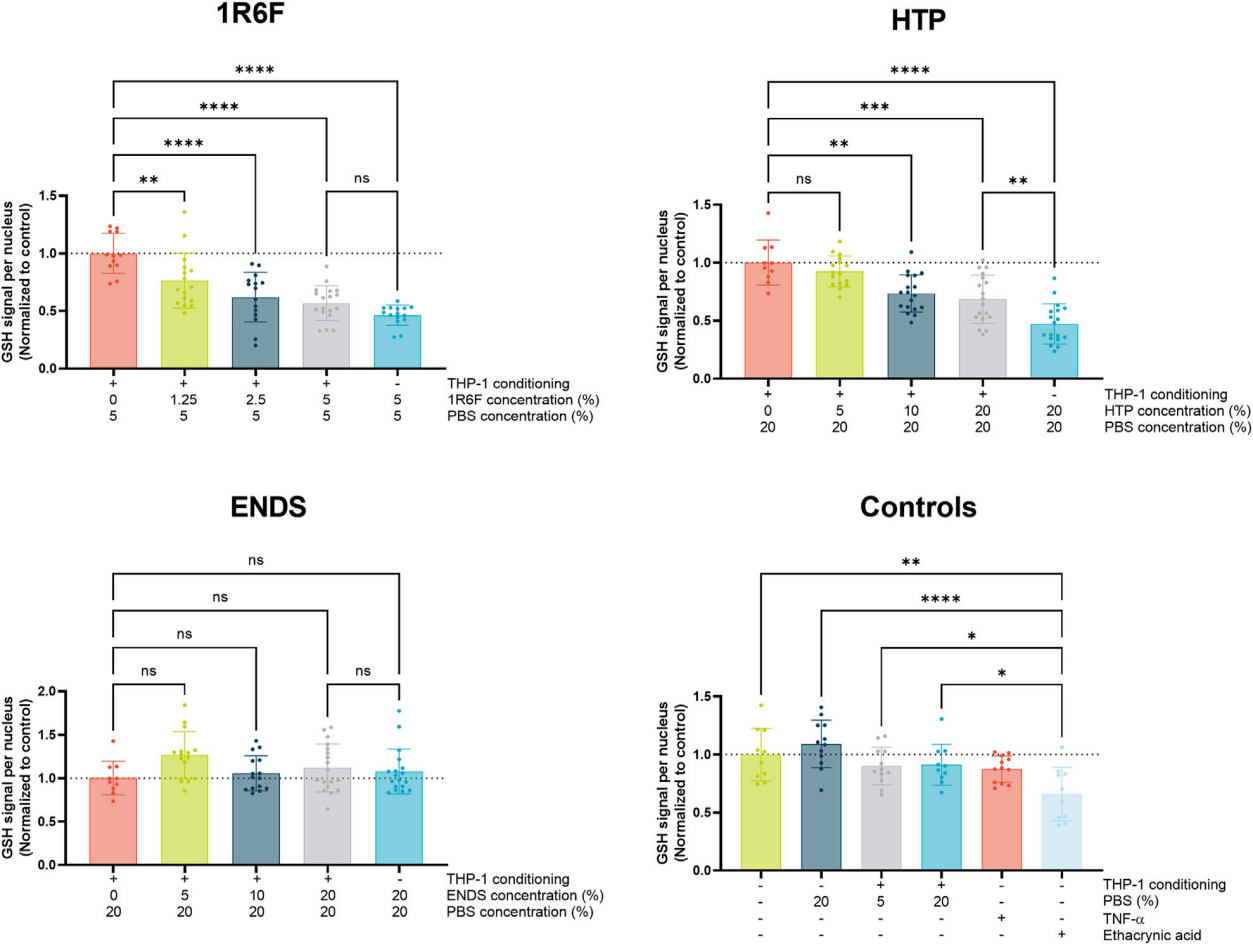
Glutathione (GSH) signal per endothelial cell nucleus (normalised to respective control values) following 4 h exposure of the model to THP-1-conditioned treated medium (treated with 1R6F, HTP and ENDS aerosol bubbled bPBS at the concentrations shown). Corresponding total PBS vehicle concentration, unconditioned medium treatments and positive controls, ethacrynic acid and TNFα are also shown. Statistical significance was determined using a one-way ANOVA. Statistical significance is marked as follows: ns = not significant, *p < 0.05, **p < 0.01, ***p < 0.001 and ****p < 0.0001.

With increasing concentrations of 1R6F extract in the pre-conditioned medium (up to 5%), a dose-dependent reduction in GSH signal was observed compared to control (Figure 3), indicating an increase in oxidative stress/species present with increasing dose. This trend was also observed for the HTP, however at four times higher PBS concentrations (4 times greater on a nicotine basis) compared to 1R6F (Figure 3). In contrast, there were no significant deviations from vehicle (PBS) for the ENDS test article (Figure 3). Upon comparison of the pre-conditioned medium treatments to the addition of the highest concentration of test article to the OrganoPlate culture only, there were further reductions in glutathione for the 1R6F and HTP samples, however this was only significant for HTP; this was not observed for the ENDS sample, however. Negative control responses were not significantly different to one another, however, positive control, ethacrynic acid elicited a significant reduction in GSH in comparison. TNF-α was also assessed, however, this did not elicit significant differences in GSH levels compared to the negative controls.

#### Monocyte adhesion

**FIGURE 4:**
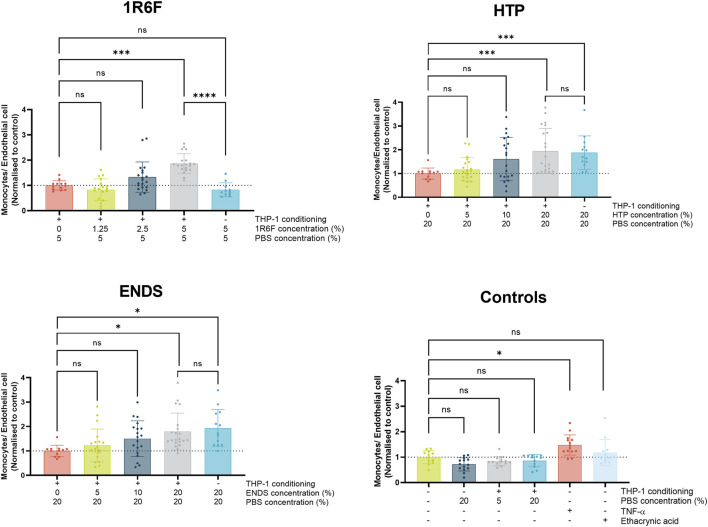
Monocyte adhesion expressed as number of monocytes per endothelial cell (normalised to respective control values) following 24 h exposure of the model to THP-1-conditioned treated medium (treated with 1R6F, HTP and ENDS aerosol bubbled bPBS at the concentrations shown). Corresponding total PBS vehicle concentration and unconditioned medium treatments are also shown. Statistical significance was determined using a one-way ANOVA. Statistical significance is marked as follows: ns = not significant, *p < 0.05, **p < 0.01, ***p < 0.001 and ****p < 0.0001.

Upon assessment of the pre-conditioned treatments, dose-dependent increases in monocyte adhesion were observed for all three test articles, however these were only significant compared to negative control (PBS) at the respective highest concentrations applied (Figure 4). Therefore, the bPBS concentrations required to induce significant responses for the HTP and ENDS was four-times that observed with 1R6F, translating to 40 and 54 μg/mL nicotine, respectively. When the test articles were added to the system in the absence of THP-1 medium conditioning, but at concentrations equivalent to the highest respective concentrations applied for each test article, outcomes were similar (i.e., significantly elevated to similar levels compared to negative control) for the HTP and ENDS test articles. In contrast, the 1R6F test article-only control induced a slight reduction in monocyte adhesion compared to control, and top concentration with the THP-1 conditioned medium. The four negative control conditions (untreated, no THP-1 conditioning + PBS, THP-1 conditioning +5% PBS, THP-1 conditioning +20% PBS) and positive control, ethacrynic acid, produced similar outcomes to one another, i.e., no significant changes in monocyte adhesion. However, compared to untreated control, the endpoint positive control, TNF-α, induced significant increases in monocyte adhesion.

#### ICAM-1 expression

**FIGURE 5:**
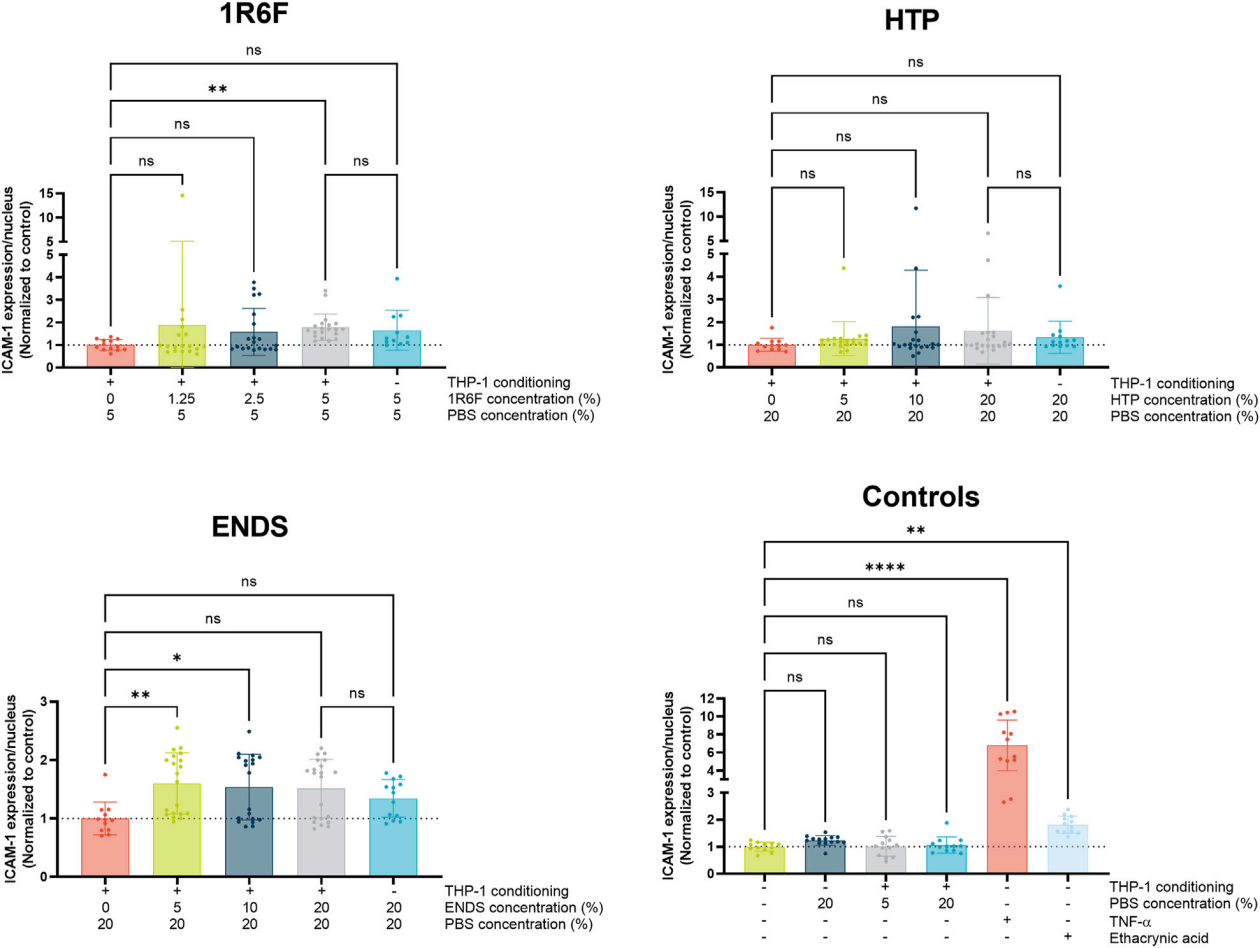
ICAM-1 expression in the endothelial cells following 24 h exposure of the model to THP-1-conditioned treated medium (treated with 1R6F, HTP and ENDS aerosol bubbled bPBS at the concentrations shown). Corresponding total PBS vehicle concentration and unconditioned medium treatments are also shown. Statistical significance was determined using a one-way ANOVA. Statistical significance is marked as follows: ns = not significant, *p < 0.05, **p < 0.01, ***p < 0.001 and ****p < 0.0001.

To assess potential mechanisms involved in monocyte adhesion, ICAM-1 expression on the endothelial cells was assessed (Figure 5). The highest 1R6F-THP-1 conditioned medium treatment induced a significant increase in ICAM-1 expression compared to PBS-THP-1 conditioned medium control. The response was also similar with the compound only control, however this was not significant compared to the PBS-THP-1 conditioned medium control. The HTP treatments did not result in any significant changes in ICAM-1 expression, although high variability was observed for the HTP-THP-1 conditioned medium concentrations. In contrast, the two lower concentrations of the ENDS-THP-1 conditioned medium treatment induced significant increases in ICAM-1 expression, which this did not reflect the monocyte adhesion outcomes observed. The four negative controls did not induce any changes in ICAM-1 expression, however, TNF-α and to a lesser extent ethacrynic acid induced significant increase in ICAM-1 expression. Overall, the results for ICAM-1 expression did not mirror the trends observed for the monocyte adhesion, and therefore other factors may be involved in this process.

#### Inflammatory markers

**FIGURE 6:**
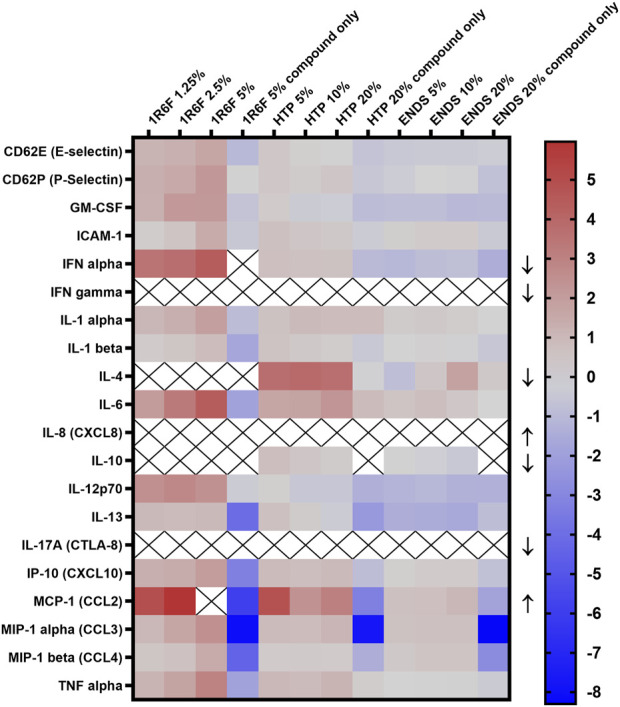
Heatmap of Inflammatory cytokine levels (log 2 transformed; normalised to respective negative control values) following exposure of the model to the respective test articles for 24 h. X indicates out of range values and arrows indicate whether the reading was above (upwards-pointing arrows) or below (downwards-pointing arrows) detection limits. ENDS: Electronic nicotine delivery system; HTP: Heated tobacco product.

A panel of 20 inflammatory mediators was assessed using the respective treatments (Figure 6). Some analyses induced signals that were either too high or low to read, indicated by ‘X’ on the diagram and an arrow denoting the direction; therefore, these analytes’ measurement require further optimisation. Upon comparison of the test article-THP-1 conditioned media treatments, 1R6F induced the overall greatest increases in the markers, followed by the HTP and then the ENDS samples (but at 4 times higher concentrations than 1R6F). Additionally, ENDS induced the most decreases in inflammatory markers, but changes were to a lesser degree (fold-change) than the increases seen for 1R6F. Dose dependence varied by marker, i.e., responses were not always linear. Compound only (non-THP-1-conditioned medium treatments) controls for all test articles resulted in reductions in inflammatory mediators, indicating a role for medium pre-conditioning using THP-1 cells in the inflammatory response.

## Discussion

This study aimed to optimise an *in vitro* cardiovascular model on-a-chip co-culture for the assessment of aqueous extracts of aerosols/smoke from three distinct nicotine delivery product categories. Following preliminary optimisation of the model using 1R6F reference cigarette smoke bPBS, aerosol bPBS generated from a *my*blu ENDS product and a Pulze and iD HTP were additionally assessed. A number of endpoints associated with atherogenesis, in addition to general model viability, were assessed following treatment of a HCAEC vessel on-a-chip with THP-1 monocyte-bPBS pre-conditioned media. These were: cell viability (1R6F), barrier integrity (1R6F), glutathione depletion, monocyte adhesion, ICAM-1 expression and inflammatory mediator readouts. Overall, 1R6F was consistently the most potent test article; with responses for the ENDS and HTP bPBS-THP-1 conditioned medium only observed at higher bPBS concentrations than 1R6F. Overall the results suggest that NGP may have reduced atherogenic potential compared to cigarettes.

### The 3D OrganoPlate model demonstrated defined responses to 1R6F reference cigarette smoke extract

To optimise the *in vitro* system for the application of nicotine product aerosol aqueous extracts, 1R6F reference cigarette smoke bPBS was initially applied to the system. Based on previous studies using endothelial-monocytic co-cultures, pre-conditioning of medium using monocytes exposed to the test articles was applied as the exposure condition within the HCAEC vessel to model indirect/secondary effects on the endothelium (Poussin et al., 2014). Based on the endothelial barrier integrity and cytotoxicity outcomes, it was concluded that 1R6F bPBS should be applied to the pre-conditioning medium up to a concentration of 5% to ensure effects in the subsequent main experiment were not related to excessive cytotoxicity in the *in vitro* model. The application of higher concentrations of PBS (i.e., 20%, 40%) was also carried out in the pre-study to assess whether addition of historically less potent NGP bPBS test articles (Simms et al., 2022; Chapman et al., 2023) could potentially be applied at higher concentrations without confounding solvent effects. Application of 40% PBS to the cell system did not appear to significantly affect cell viability, however some decrease in barrier integrity was observed compared to 20% PBS. In addition to this, PBS was also applied to THP-1 monocyte cultures for 2 h up to a concentration of 40% to assess the maximal concentration for the medium pre-conditioning step (Supplementary Figure S1). At 40%, although not significantly altered compared to untreated control, there was an increase in variability between replicates. Therefore, the maximal concentration of PBS to be applied for the additional test articles in the main study (HTP and ENDS aerosol bPBS) was selected at 20%.

### Pre-conditioning of medium with THP-1 and the test articles elicited defined responses

To increase the model’s human/*in vivo* relevance, and better represent potential inflammatory responses/atherogenic processes, pre-conditioned medium was generated with THP-1 monocytes for the exposure within the HCAEC vessels (Poussin et al., 2020). To confirm whether the pre-conditioned media played a role in the responses observed, direct bPBS exposure to the HCAEC vessel treatment conditions were also included at the highest respective bPBS concentrations applied for each test article. The direct addition of the test articles only appeared to further decrease GSH levels within the OrganoPlate system 4 h following the start of exposure for the 1R6F and HTP highest concentrations, however this was not the case for the ENDS treatments, which were not different to one another. This could potentially be attributed to the presence of modulators within the pre-conditioned medium regulating some of the endothelial response to the 1R6F and HTP bPBS extracts present, or initial maximal (nominal) exposure to reactive species within the 1R6F/HTP bPBS. This effect, however, was not observed for the ENDS and HTP bPBS in the monocyte adhesion endpoint, although pre-conditioning demonstrated a clear influence on THP-1 adhesion in the 1R6F bPBS-exposed model. Upon comparison of the effects of exposures including pre-conditioned medium to test-article-only exposures on the inflammatory panel readouts, there was a clear dose-dependent influence of pre-conditioned medium within the system, with mainly increases observed for 1R6F and the HTP, and only slight increases or decreases for the ENDS. In contrast, across all test articles, levels of the inflammatory modulators largely decreased for the compound only controls, again with a trend for the greatest changes with 1R6F, then HTP, then ENDS. This may explain some of the differences in responses observed in the GSH/monocyte adhesion endpoints in the presence and absence of pre-conditioning. These results highlight the importance of capturing the interaction of different cell types within *in vitro* models.

### Distinct oxidative stress responses were achieved upon comparison of the test articles

GSH depletion was used as a proxy assessment for the presence of oxidative stress following exposure of the co-culture to test article pre-conditioned media. A reduction in GSH signal correlates with a response to the presence of oxidative species, which may be a direct result of constituents of the test articles or secondary inflammatory responses (Poussin et al., 2016; Yang et al., 2017). As anticipated, 1R6F demonstrated the most potency out of the three test articles, inducing dose-dependent reductions in GSH, and at concentrations four times lower than those inducing a similar response for the HTP test article. In contrast, the ENDS sample did not induce any significant reductions in GSH at the concentrations tested. Oxidative stress can drive the expression of adhesion molecules in the vascular endothelium in the early stages of atherosclerosis, leading to the recruitment and adhesion of monocytes (Björkegren and Lusis, 2022; Ohara and Ito, 2023); the oxidative stress outcomes under pre-conditioned medium treatments indeed appeared to be reflected by the monocyte adhesion assay outcomes for 1R6F and the HTP, however, not for the ENDS, suggesting the role of potential other factors here.

### Assessment of the factors involved in monocyte adhesion

Monocyte adhesion is a key step in the development of atherosclerosis: monocytes attach to the vascular endothelium and migrate into the vessel intima where they can transform into macrophages (Fearon et al., 2013). These macrophages in turn form foamy macrophages following uptake of oxidised LDL, and accumulation of these fatty cells contributes to the formation of plaques (Javadifar et al., 2021). In order to model the early phase of this (monocyte adhesion), FITC labelled THP-1 monocytes were perfused through the vessels for 30 min at the end of the main (24 h) exposure. All three test articles induced apparently dose-dependent increases in monocyte adhesion following exposure, however, in light of replicate variability, this was only significant at the highest dose tested for each respective test article. For the highest concentrations tested, compared to treatments without pre-conditioned medium, there were no significant differences in monocyte adhesion for the ENDS and HTP bPBS, suggesting that exposure of the endothelium to the test article constituents may be the main driver for monocyte adhesion. However, when making this comparison for 1R6F, the presence of pre-conditioned medium was required to induce significant increases in monocyte adhesion, which was not observed in the absence of pre-conditioning. This may be due to the different compositions of 1R6F bPBS and the NGP bPBS samples, which may elicit different response mechanisms, for example, higher levels of nicotine within the NGP samples; this is reflected in the inflammatory marker outcomes. In comparison to similar studies, the magnitude of change in monocyte adhesion differed from that observed by others (Poussin et al., 2020; Ohashi et al., 2023), however, this could be due to methodological differences in detecting and quantifying the cell signal, or differences in chemical fractions applied. For example, Ohashi et al. (2023) utilised total particulate matter (TPM)/aerosol collected mass (ACM), fractions of cigarette smoke/HTP aerosol respectively, whereas this study applied aqueous smoke/aerosol extracts. Furthermore, whilst both are human primary monocyte models, there might be differences between the MM6 cell line used by Poussin et al. (2020), and the THP-1 cell line used in this study in their responses to the test articles.

ICAM-1 is known to be associated with adhesion of monocytes to the vascular endothelium during the atherogenic process (Lawson and Wolf, 2009; Ohara and Ito, 2023). Therefore, to understand potential drivers for monocyte adhesion within the co-culture following exposure to the respective test articles, measurement of endothelial ICAM-1 expression was carried out. For the HTP bPBS, endothelial ICAM-1 expression did not appear to significantly change upon exposure of the test system to the test articles/pre-conditioned medium, indicating that, where monocyte adhesion was observed, ICAM-1 was not a major driver for these effects. Monocyte adhesion is also linked to other adhesion molecules, including VCAM-1 (Edwards and Thomas, 2017), which was not measured in this study, but could warrant assessment in future investigation. The present findings are consistent with those of others’, where ICAM-1 expression was found not to always directly correlate with monocyte adhesion (Poussin et al., 2018); the study additionally found that VCAM-1 expression decreased upon exposure of cardiovascular cells to similar test articles, and proposed other adhesion molecules may be involved (Poussin et al., 2018). Further to this, many other inflammatory mediators, including those measured within the inflammatory panel applied within this study, are linked to monocyte adhesion (Poussin et al., 2014; Kong et al., 2022), and therefore there could be some involvement of these in the responses observed in this study, as discussed in the next section. Interestingly, for the ENDS bPBS, endothelial ICAM-1 expression was significantly increased at the two lower dose levels tested (5% and 10% bPBS), but not for the highest treatment concentration (Figure 5). This could, however, be due to high variability within the test replicates; comparison to the ICAM-1 readout for the ENDS bPBS in the inflammatory marker panel (Figure 6) indicated low ICAM-1 expression at the three concentration levels applied (pre-conditioning condition). 1R6F did induce significant increases in endothelial ICAM-1 expression at the highest concentration tested (5%), however, again, high variability was observed for the outcomes. Therefore, ICAM-1 may be involved in the monocyte adhesion observed for 1R6F, which is supported by the outcomes for the inflammatory panel.

### Distinct inflammatory marker profiles were observed for each test article

As mentioned above, the three test articles induced distinct dose-dependent inflammatory profiles compared to one another, with 1R6F eliciting the greatest increases in markers, followed by lesser responses to the HTP, and even lesser changes compared to vehicle control for the ENDS test article. There was additionally a clear distinction between readouts for the test article-only treatment compared to the pre-conditioning treatments, highlighting the cross-talk of different cell types in cellular responses to exogenous agents, and the role of secondary responses to exposure. A number of the markers within the panel play a role in processes involved in the early stages of atherosclerosis, including drivers of immune cell recruitment, adhesion and migration and modulators of other stress responses (Libby et al., 2002; Poussin et al., 2014; Fatkhullina et al., 2016). The dose-dependent increases induced by the 1R6F bPBS following the 24 h pre-conditioning were largely consistent across the markers measured, however, IL-4 and IL-10 levels were below detection limits for all three treatment levels. Due to the interplay of different mediators, this downregulation may have been driven by the increased levels and feedback of other cytokines present, or potentially linked to the absence of stimulation of IL-10 by IL-4 (Mitchell et al., 2017), which may represent a lack of presence of driver for this kind of cytokine response. Many of the markers within the panel have known roles in cellular recruitment (E-selectin, P-selectin, ICAM-1, etc.) and additionally drivers of the process such as TNF-α (Warboys et al., 2011; Fatkhullina et al., 2016); whilst there were the clear dose-dependent trends for 1R6F in the pre-conditioned exposure conditions, this was not always the case for the NGP exposures. However, dose-dependent increases in mediators such as TNF-α and ICAM-1 mirror the outcomes in the oxidative stress and monocyte adhesion assays for the HTP. Interestingly, where levels were below detection limits for IL-4 for 1R6F, levels were elevated for all three HTP treatment concentrations, indicating that different inflammatory mechanisms may be underway in response to the discrete product categories. The inflammatory panel revealed greater dose-dependent trends with ICAM-1 than seen in the endothelial assessment, indicating that variability may have driven a lack of significant responses or that there is no correlation between the membrane-bound and the soluble forms of ICAM-1 (Theresa et al., 2014). However, the ICAM-1 outcomes for ENDS was reflected in the inflammatory panel readout, where levels were increased at the lower two test concentrations only. Overall, the panel indicated a complex interplay of inflammatory mediators present in the cell culture medium, which was both dose- and test article-dependent. Additionally, the panel only assessed 20 markers, and others have demonstrated further mediators of atherogenic endpoints in response to nicotine delivery products (Poussin et al., 2018; Poussin et al., 2020), therefore, further characterisation, such as transcriptomics analysis, may be beneficial.

### The distinct test articles demonstrated different dosimetry

Upon comparison of the three test products applied within this study, the 1R6F reference cigarette, an ENDS and an HTP, there were overall clear distinctions between responses. Firstly, the highest bPBS concentration applied to the *in vitro* system was 5% for 1R6F due to losses in cell viability and barrier integrity at the concentrations tested above this (10% and 20%) in the initial dose-finding assessments (Figure 2A,B). In contrast, the two NGP products were able to be applied to the test system up to a concentration of 20% and generally this 4x higher bPBS concentration was required to elicit significant responses to the NGP test articles. These outcomes were associated with the relative numbers and levels of carbonyls measured in the respective bPBS samples (Table 3); the relative potencies of the test articles in the *in vitro* system was 1R6F >> HTP > ENDS. For the ENDS bPBS, levels were below LOQ for seven carbonyls and it contained 26-fold less formaldehyde than the 1R6F stock. Overall, there were substantial reductions in carbonyls within the HTP bPBS compared to 1R6F. Where responses were observed for the NGP, potential drivers may be product/extract dependent. This corresponds to the temperatures used to generate the respective smoke/aerosols: cigarettes burn tobacco whereas the HTP heats tobacco at 345^o^C, and a combustion process and generation of the associated chemicals therefore does not take place (Chapman et al., 2023). Furthermore, lower temperatures still are used to heat e-liquids in ENDS (Jaegers et al., 2021; Li et al., 2021), although differences in e-liquid and tobacco compositions will also have an influence on aerosol characteristics. When calculated on a relative nominal carbonyl level applied to cell culture medium within the bPBS, levels were comparable between the 5% 1R6F bPBS and 20% HTP bPBS. This provides a potential explanation, coupled with the higher nicotine concentrations present in the HTP bPBS, for generally similar magnitudes of responses in some of the endpoints measured. Nicotine is known to have toxic *in vitro* effects at high concentrations (Rudd et al., 2020) and this may also have been a driver for effects in the case of the ENDS bPBS, which demonstrated the highest relative levels of nicotine. However, 1R6F still demonstrated greater potency, suggesting other toxicants may be present within this sample, either direct-acting, or those causing secondary effects; further analysis of all bPBS samples would be the next step to understand the drivers for the relative responses observed to the test articles in more detail. An additional limitation in defining the relative exposures is the difficulty in accurately capturing the levels of each component of the extract due to its potential degradation within the pre-conditioning medium/loss upon removal of the THP-1 cells. It is also of note that nicotine concentrations applied within the *in vitro* system ranged from 2.3 (1R6F) - 53.64 (ENDS) μg/mL (Table 1), concentrations which greatly exceed the 5–50 ng/mL range observed in the blood plasma of smokers, HTP and EVP users (Benowitz et al., 2009; Jacobson et al., 2020; McDermott et al., 2023) following single product use; it can also be inferred that this kind of difference would be the case for other compounds present within the smoke/aerosols of the products. However, it must be noted that use of multiple nicotine products during the day may increase nicotine exposure and is product and user topography dependent, for example, as seen with ENDS (Jacobson et al., 2020). A recent *in vitro* to *in vivo* extrapolation study by Moreau et al. (2024) predicted that due to rapid nicotine elimination, following the use of 10 cigarettes or HTP sticks, a steady state of nicotine is still only approximately double that observed following the use of one product. Therefore, the responses observed in this study are still to supraphysiological levels of nicotine exposure, the study has demonstrated that the co-culture system is responsive to nicotine delivery product extracts, and may act as a useful tool in screening for effects at physiological levels in future. These levels are also regularly applied for *in vitro* assessments of such products (Czekala et al., 2021; Chapman et al., 2023).

### Limitations and future directions

This study has provided valuable insights into the effects of three discrete nicotine delivery products in an *in vitro* 3D co-culture cardiovascular model. However, this study is not without its limitations. First, whilst the endpoints modelled some processes involved in atherosclerosis development, there are many other factors and key molecular events involved, which develop over a number of years (Libby et al., 2002; Rafeieian-Kopaei et al., 2014). Whilst it would be difficult to fully model *in vitro*, the investigation of further endpoints associated with atherosclerotic processes may add to the weight of evidence for the tobacco harm reduction potential of the NGP provided by this study. These include further investigation markers involved in monocyte adhesion, assessment of immune cell migration and additionally assessment of the formation of foamy macrophages (Park, 2021; Riddle et al., 2022). Ideally, human primary monocytes would be used for these experiments instead of the THP-1 monocytic cell line since, although a convenient and valuable tool to study monocyte function, there are significant differences between the immortalized cell line and the primary cells they are representing (Bosshart and Heinzelmann, 2016).

Furthermore, whilst the inflammatory panel provided useful insights into the dose-dependent effects of the test articles (and a comparison of both the pre-conditioning and test article-only conditions), some of the markers produced signals above or below the limits of detection, and therefore further optimisation may be required for these analytes.

The study also only assessed one variant of each of the NGP products, so does not account for changes in composition or flavour between products. However, the evidence indicates that each discrete product category generally induces relative responses as observed here: cigarette is the most potent test article, and responses are reduced upon exposure to HTP and further still with ENDS products (McNeill and Munafò, 2013; Zeller, 2019; Simms et al., 2022). An aqueous aerosol extract was selected for application in this study; this is a medium that has been previously utilised for the *in vitro* assessment of nicotine delivery products, and particularly within these types of models (Poussin et al., 2020; Simms et al., 2020; Czekala et al., 2021) and may model the aqueous soluble fraction of smoke aerosol, which may most likely absorbed through the lung alveolar wall and to be circulating within the blood (Poussin et al., 2014). However, comparison of effects with the addition of filterpad trapped particulate matter/aerosol collected mass may be beneficial to highlight any differences in results with the addition of this fraction of smoke/aerosol. Furthermore, the test articles in the study were not fully characterised in terms of their chemical composition (only select analytes within the bPBS stock solutions were measured) and therefore exposure comparisons were based on the stock solution dilutions rather than actual measurements within the chip. Additionally, the effects of primary constituents of the stock solutions, and not necessarily physiologically relevant metabolites, were assessed here. Therefore, further characterisation of exposure levels and metabolism of the test articles over time and also maintenance of initial exposure levels within the system would be useful to assess outcomes under even more physiologically relevant conditions.

## Conclusion

Overall, this study has provided initial validation of an *in vitro* 3D cardiovascular co-culture model to assess the effects of various inhaled nicotine product aerosol aqueous extracts on endpoints related to atherosclerosis development. Across the endpoints tested, the 1R6F reference cigarette consistently displayed increased potency than the NGPs, with the HTP demonstrating slightly greater effects than the ENDS product. These outcomes could be attributed to the distinct differences in the levels and numbers of carbonyls. Through inference other potential toxicants, present within the NGP bPBS; levels were greatest for 1R6F, followed by substantial reductions in levels for the HTP and largely below LOQ values for the ENDS extract. The study also provided valuable insights into the specific NGP included in the study, which have not been previously characterised alongside each other in such a model. The study additionally provided further information to that already published on such models, including the assessment of a greater range of inflammatory mediators than previously assessed (Ohashi et al., 2023). Overall, this study substantiates existing evidence that NGP can provide a potentially reduced harm form of nicotine delivery in the context of atherosclerosis for adult smokers who do not wish to quit nicotine.

## Data Availability

The original contributions presented in the study are included in the article/Supplementary Material, further inquiries can be directed to the corresponding author.

## Supplementary material

SUPPLEMENTARY FIGURE S1 Effects on cell viability (WST-8 assay) of a 2 hour exposure of THP-1 monocytes to phosphate buffered saline (PBS) solution in a pre-study assessment.

## Abbreviations

ANOVA, Analysis of variance; bPBS, Bubbled phosphate buffered saline (solution); ECM, Extracellular matrix; ENDS, Electronic nicotine delivery system; GSH: Glutathione; HBSS, Hank’s Balanced Salt Solution; HCAEC, Human Coronary Artery Endothelial Cells; HCI, High content imaging; HEPES, N-(2-Hydroxyethyl)piperazine-N′-(2-ethanesulfonic acid); HPHCs, Harmful and potentially harmful constituents; HTP, Heated tobacco product; ICAM-1, Immunoglobulin cell adhesion molecule-1; ISO, International Organization for Standardization; LOQ, Limit of quantification; NGP, Next-generation nicotine delivery product; ns, Not significant; Papp, Apparent permeability; PBS, Phosphate buffered saline (solution); RPMI, Roswell Park Memorial Institute (cell culture medium); THR, Tobacco harm reduction; TRITC, tetramethylrhodamine isothiocyanate; VCAM-1, Vascular cell adhesion molecule-1; WST-8, Water-soluble tetrazolium salt.
