# Supplementary figures and images for: Optimisation of an in vitro human cardiovascular model on-a-chip for toxicological assessment of nicotine delivery products
Source: Front Toxicol. 2024 Jun 13;6:1395670. doi: 10.3389/ftox.2024.1395670 (PMC11208624; doi:10.3389/ftox.2024.1395670)

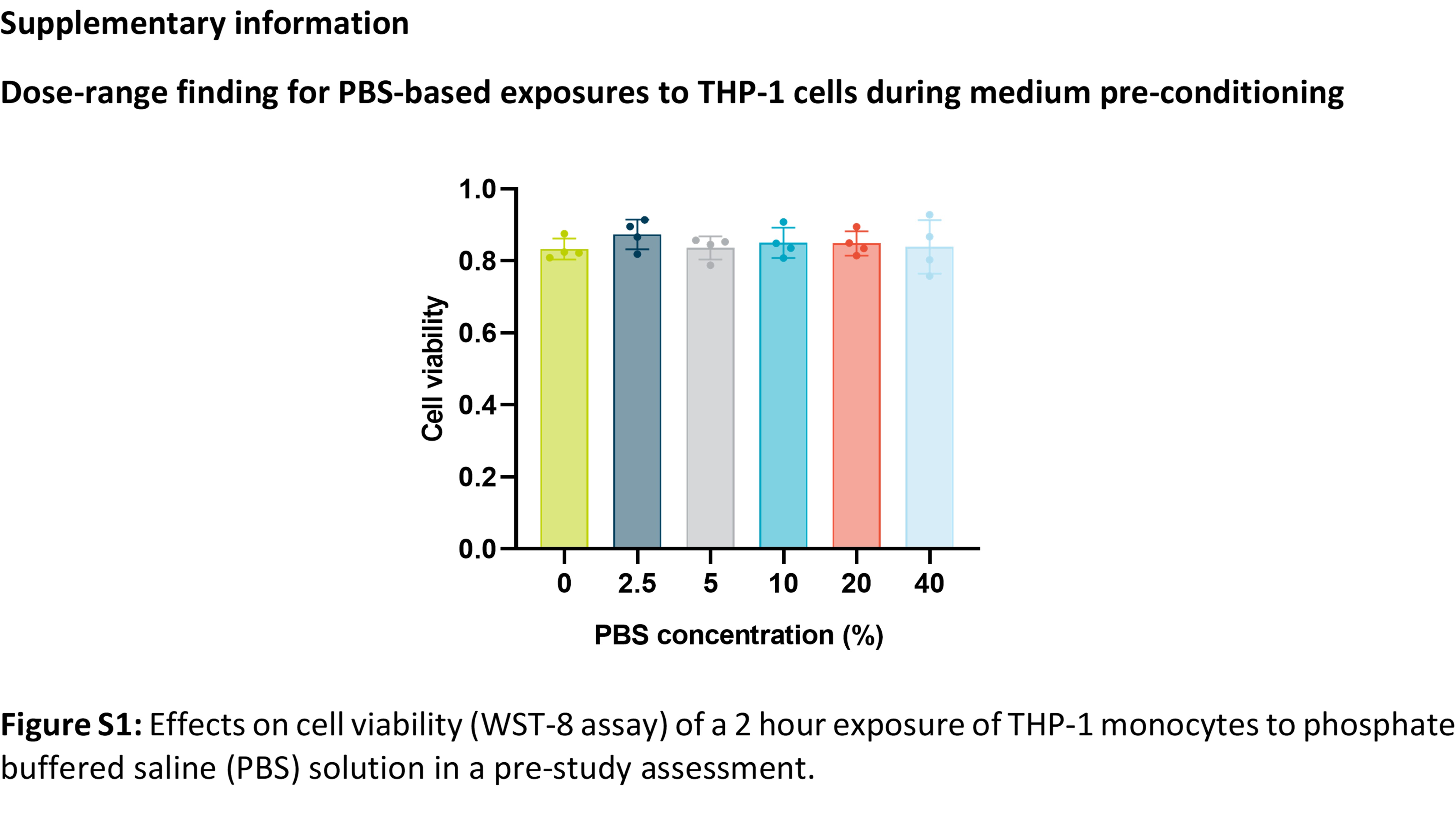

Supplement: Supplementary file 1 [file Image1.jpg]
